# Simultaneous Measurement of Neuronal Activity in the Pontine Micturition Center and Cystometry in Freely Moving Mice

**DOI:** 10.3389/fnins.2019.00663

**Published:** 2019-06-25

**Authors:** Jiwei Yao, Qianwei Li, Xianping Li, Han Qin, Shanshan Liang, Xiang Liao, Xiaowei Chen, Weibing Li, Junan Yan

**Affiliations:** ^1^Department of Urology, Southwest Hospital, Third Military Medical University, Chongqing, China; ^2^Brain Research Center and State Key Laboratory of Trauma, Burns, and Combined Injury, Third Military Medical University, Chongqing, China; ^3^Department of Urology and Nephrology, The Third Affiliated Hospital, Chongqing Medical University, Chongqing, China

**Keywords:** the optical fiber-based Ca^2+^ recording, cystometry, pontine micturition center, urination, freely moving mice

## Abstract

Understanding the complex neural mechanisms controlling urinary bladder activity is an extremely important topic in both neuroscience and urology. Simultaneously recording of the bladder activity and neural activity in related brain regions will largely advance this field. However, such recording approach has long been restricted to anesthetized animals, whose bladder function and urodynamic properties are largely affected by anesthetics. In our recent report, we found that it is feasible to record bladder pressure (cystometry) and the related cortical neuron activity simultaneously in freely moving mice. Here, we aimed to demonstrate the use of this combined method in freely moving mice for recording the activity of the pontine micturition center (PMC), a more difficultly approachable small region deeply located in the brainstem and a more popularly studied hub for controlling bladder function. Interestingly, we found that the duration of urination events linearly correlated to the time course of neuronal activity in the PMC. We observed that the activities of PMC neurons highly correlated with spike-like increases in bladder pressure, reflecting bladder contractions. We also found that anesthesia evoked prominent changes in the dynamics of the Ca^2+^ signals in the PMC during the bladder contraction and even induced the dripping overflow incontinence due to suppression of the neural activity in the PMC. In addition, we described in details both the system for cystometry in freely moving mice and the protocols for how to perform this combined method. Therefore, this work provides a powerful approach that enables the simultaneous measurement of neuronal activity of the PMC or any other brain sites and bladder function in freely behaving mice. This approach offers a promising possibility to examine the neural mechanisms underlying neurogenic bladder dysfunction.

## Introduction

Neural circuits integrated at different levels of the central and peripheral nervous systems tightly control normal bladder function (Fowler et al., 2008; Benarroch, 2010; de Groat et al., 2015). Injuries or diseases of the nervous system may lead to neurogenic bladder (NGB) dysfunction (Fowler et al., 2008; Harris and Lemack, 2016). NGB is generally found in patients with neurological disorders, and affects their quality of life (Ginsberg, 2013). However, our current insights into NGB are very limited. A better understanding of the neural mechanisms controlling bladder would provide new strategies for the treatment of NGB.

The pontine micturition center (PMC) is a small brainstem nucleus located in the caudal pontine tegmentum near the locus coeruleus (LC) (Verstegen et al., 2017). The PMC is suggested to be necessary for urination in animals and humans (Valentino et al., 2011). Inhibition or lesion of the PMC causes urinary retention and prevents urination, and electrical or optogenetic stimulation in this region drives bladder contraction and triggers urination (Sugaya et al., 1987; Sugaya, 1988; Hou et al., 2016; Yao et al., 2018). Projections of PMC neurons directly go to the sacral spinal cord preganglionic bladder motor neurons (Blok and Holstege, 1998; Verstegen et al., 2017). PMC neurons integrate the pro- or anti-urination signals (Drake et al., 2010; de Groat and Wickens, 2013; Hou et al., 2016) and receive converging inputs from multiple upstream brain regions (Hou et al., 2016). Therefore, PMC neurons are an important entry point for investigations of the larger-scale brain network of bladder control.

Simultaneous measurement of neural activity and cystometry is a potentially useful approach to examine the neural mechanisms that control bladder functions (Manohar et al., 2017). Many classic studies used electrodes to record correlating neuronal responses in the PMC together with bladder pressure measurement in anesthetized or restrained animals (Willette et al., 1988; Tanaka et al., 2003). Data from traditional electrophysiology are limited by the low stability of long-lasting recordings and the lack of cell-type specificity (Guo et al., 2015). Anesthesia suppresses bladder function and affects the urodynamic results (Aizawa et al., 2015; Schneider et al., 2015). However, an efficient approach to combine the monitoring of neuronal activity with cystometry in freely moving animals has long been lacking. In our recent report, we demonstrated the feasibility of the method for recording both bladder pressure and neuronal activity of motor cortex in freely moving mice (Yao et al., 2018), but whether this method can be extended to a more difficultly reachable region, the PMC, is unclear. This is an important attempt, as the PMC plays important yet many remaining unknown functions in controlling bladder and urination, as mentioned above.

The development of genetically encoded Ca^2+^ indicators (GECIs) (Grienberger and Konnerth, 2012) and genetically targeted techniques has simplified the use of optical fiber-based Ca^2+^ recording (also known as fiber photometry). Fiber photometry is currently a popular choice for the monitoring of cell type-specific neuronal activities of any brain areas in freely behaving animals (Stroh et al., 2013; Gunaydin et al., 2014). This approach is suitable for the detection of the average spiking activity of a cluster of neurons (Adelsberger et al., 2005) or of their axon terminals (Qin et al., 2018), and the general consensus is that neuronal Ca^2+^ transients reflect neuronal action potential firing (Kerr et al., 2005). Fiber photometry costs less, and data acquisition and analyses are much simpler than electrophysiology (Cui et al., 2014). Therefore, we developed a simple method using fiber photometry to monitor the neuronal activity of PMC neurons together with cystometry in freely moving mice.

## Materials and Methods

### Animals

Wild-type adult C57/BL6J mice (both sexes, aged 3–4 months old) were used in all experiments and purchased from the Laboratory Animal Center at the Third Military Medical University. Mice were socially housed under a 12-h light/dark cycle and provided with *ad libitum* food and water. Each mouse implanted with a PE10 catheter insertion into the bladder or an optic fiber into the brain was individually housed and habituated to the experimental device prior to recording. The Third Military Medical University Animal Care and Use Committee approved all experimental procedures, which were performed in accordance with institutional animal welfare guidelines.

### *Trans*-Synaptic Tracing

Mice were anesthetized via an intraperitoneal injection (i.p.) of 1% sodium pentobarbital (10 ml/kg). Their bladders were exposed and exteriorized through the middle abdominal incision. Two injections approximately 0.5 mm deep were performed across the bladder wall by use of a small glass micropipette attached to a microsyringe (10 μl). PRV-EGFP (1.71 × 10^8^ PFU/ml in 1 μl) was injected at each site at a speed of 200 nl/min via a syringe pump. The micropipette was held in each injection site for 5 min. Each bladder was returned to the mouse abdominal cavity, and finally the abdominal wound was closed. Mouse brains were removed at 4.5 to 5 days after PRV-EGFP injection and stored in fixative solution prior to sectioning.

### Viral Injection

Mice were placed in a stereotaxic frame (RWD Technology Co., Ltd., China) with a heated pad and anesthetized with 1–1.5% isoflurane in oxygen. A scalp incision was performed using eye scissors. A craniotomy (1 mm × 1 mm) was performed using a dental drill above the PMC (AP: −5.45 mm; ML: 0.70 mm; DV: −3.15 mm). AAV-hSyn-GCaMP6f (AAV2/9, titer: 2.59 × 10^12^ viral particles/ml, Obio Biotechnology Co., Ltd., China) or AAV-hSyn-EGFP (AAV2/8, titer: 1.63 × 10^13^ viral particles/ml, Obio Biotechnology Co., Ltd., China) was delivered using a small glass micropipette with a tip diameter of ∼20 μm. The micropipette was slowly inserted to a depth of 3.15 mm (from the brain surface), and a total volume of 80–100 nl AAV-hSyn-GCaMP6f or AAV-hSyn-EGFP was injected into the PMC. The micropipette was held in each injection site for 10 min after injection and retracted. The scalp incision was closed. Each mouse was returned to the home cage and allowed to recover for at least 3 weeks.

### Fiber Photometry Setup

A custom-built fiber photometry setup (Figure 2A) was used in this research to record population neuronal Ca^2+^ signals (Adelsberger et al., 2005; Li et al., 2017; Zhang et al., 2017; Qin et al., 2018; Yao et al., 2018). For excitation of the fluorescent genetically encoded Ca^2+^ indicator (GCaMP6f), the excitation light (the wavelength of 450–490 nm) was delivered by a light-emitting diode (LED). The accurate adjustment of the beam intensity of LED was driven by the current. Then the beam was deflected by a dichroic mirror (Di02-R488, Semrock, United States) and focused into the end of an optic fiber (200 μm diameter, NA 0.48, Doric lenses, Quebec City, QC, Canada) through a collimator. The beam intensity at the tip of the optic fiber was about 0.22 mW/mm^2^. The emitted GCaMP6f fluorescence (green) was guided back through the same fiber and then was separated from excitation blue light using the same dichroic mirror with a bandpass emission filter (FF01-535/22, Semrock, United States). Finally, the GCaMP6f fluorescence emission was monitored using an avalanche photodiode (Si APD, S2382, Hamamatsu Photonics K.K., Japan). The collected GCaMP6f fluorescence signals were digitized with a sampling frequency of 2000 Hz by the use of a multifunction I/O device (USB-6002, National Instruments, United States). Data acquisition was implemented by customized software written in the LabVIEW platform (National Instrument, United States).

### Optical Fiber Recording *in vivo*

Optical fiber recordings in the PMC were performed similarly as described previously (Adelsberger et al., 2005). Mice were anesthetized with 1–1.5% isoflurane and put on a stereotactic head frame. A scalp incision was performed using eye scissors. The craniotomy (1 mm × 1 mm) was performed using a dental drill above the PMC. An optic fiber (200 μm diameter, NA 0.48, Doric lenses, Quebec City, QC, Canada) was glued into a short cannula (ID. 0.51 mm, OD. 0.82 mm), inserted through the craniotomy and advanced slowly to 3.05 mm below the brain surface. The optic fiber was secured to the mouse skull using dental cement. Mice implanted with an optic fiber were allowed to recover for 3–5 days prior to urination behavior testing. To avoid breakage of the optic fiber in freely moving mouse, the changes in the curvature of the fiber were checked every 6 h by the experimenters and the fiber was held loosely above each mouse head at a distance of 50–80 cm. During the recordings, the optic fiber was connected to the fiber photometry system directly without using the commutator. For awake freely moving recordings, each mouse was put in a chamber with a glass bottom. Neuronal Ca^2+^ transients and behaviors were recorded simultaneously. For anesthetized simultaneous recordings, the mice were put on a heated pad under the anesthetized condition. The Ca^2+^ transients were recorded using customized acquisition software based on Labview platform (National Instrument, United States). A camera (Aigo AHD-X9, China) was used to record mouse urination events at 30 Hz under the spatial resolution of 1280 × 720 pixels. The first drop from the urethral orifice represented the onset of urination events.

### Cystometric Measurement *in vivo*

Mice were placed on a heated pad and anesthetized with 1–1.5% isoflurane in oxygen. Each bladder dome was exposed and exteriorized through a middle abdominal incision. A 7 cm PE10 tube was used as the catheter and inserted into the bladder. A loose tie of monofilament suture (6-0) was used to secure the catheter in the bladder. Then the end of the catheter was pulled out through an incision at the back of mouse neck. For testing the seal and patency of the PE10 tube in the bladder, a syringe (1 ml) connected with 30-gauge needle was attached to the end of the catheter, and the bladder was slowly filled with saline until a drop appearing at the urethral orifice to indicate that the cystometry surgery was successful. The abdominal wound and neck incision were closed, and each mouse was allowed to recover for at least 5 days prior to cystometry recordings. Before cystometry recordings in an awake or anesthetized mouse, a pressure transducer (YPJ01H; Chengdu Instrument Factory, China) was attached to a multi-channel physiological recording and signal processing setup (RM6240; Chengdu Instrument Factory, China) for bladder pressure recording. A 3-way tab was used to connect an infusion pump (RWD404; RWD Technology Corp., Ltd., China), the pressure transducer and appropriate adapter. The end of the adapter was connected with a PE50 tube. The catheter in the bladder of each mouse was slid into the end of the PE50 tube. Before each cystometric measurement, the pressure transducer was calibrated. Cystometry recordings were performed in awake mice with the normal saline infusion into the bladders via the catheter at a constant speed (10–50 μl/min). For anesthetized simultaneous recordings, the mice were anesthetized with 2.5% isoflurane firstly and then these mice were administered with urethane (1.2 g/kg, i.p.). The normal saline was infused into the bladders via the catheter at a constant speed (30 μl/min) under the anesthetized condition. Continuous urodynamic curves were recorded using a commercial acquisition software (Chengdu Instrument Factory, China).

### Immunohistochemistry

Coronal brain slices of 40 μm were obtained using a freezing microtome (Leica) and collected in 0.1 M phosphate-buffered saline (PBS) solution. Brain slices were washed in PBST (1% Triton in 0.1 M PBS) for 40 min. A blocking solution (10% donkey serum, 0.2% Triton in 0.1 M PBS) was applied for 2 h at room temperature. Brain sections were incubated with primary antibodies (chicken anti-GFP, 1:200, Abcam, ab13970; rabbit anti-tyrosine-hydroxylase, 1:200, Millipore, AB152) overnight at 4°C followed by secondary antibodies for 2 h at room temperature. The secondary antibodies used were donkey anti-rabbit conjugated with Alexa Fluor 568 (1:200, Invitrogen, A10042) and donkey anti-chicken CF488 (1:200, Sigma-Aldrich, SAB4600031). Brain slices for DAPI staining were incubated with DAPI (1:1000, Sigma) for 20 min. Finally, these slices were mounted on coverslips and imaged. Images were obtained by the use of a scanning confocal microscope (TCS SP5, Leica) and LAS AF software (Leica).

### Data Analysis

All Ca^2+^ transients were collected at the sampling frequency of 2000 Hz and low-pass filtered using the Butterworth filter. For both awake and anesthetized recordings, the relative fluorescence changes Δf/f = (f − f_baseline_)/f_baseline_ were calculated for Ca^2+^ transients, where f_baseline_ was the baseline fluorescence level taken during the current recording period during urination behavior. If the amplitude of a Ca^2+^ transient was larger than three times the standard deviation of the noise band, it was regard as a signal. The software for data analysis was custom-written on the MATLAB (MathWorks, United States)^1^. The Ca^2+^ signals were divided into 10 segments and we randomly assigned these segments with urination events or cystometry data, in order to shuffle the fiber photometry data. The duration of each urination event was measured from the urination onset (the first drop of urine appearing at the urethral orifice) to the urination offset (no drop of urine appearing at the urethral orifice).

### Statistical Analysis

Each values in this paper is represented as the mean ± s.e.m. Statistical analyses and cross-correlation analyses were calculated in MATLAB (MathWorks, United States). *P*-values for the comparison of paired data were performed using Wilcoxon signed-rank test. *P*-values for comparison of two independent samples were performed using Wilcoxon rank-sum test. ^∗∗∗^*P* < 0.001, ^∗∗^*P* < 0.01, ^*^P < 0.05; ns, no significant difference.

## Results

### Verification of PMC Location

**FIGURE 1:**
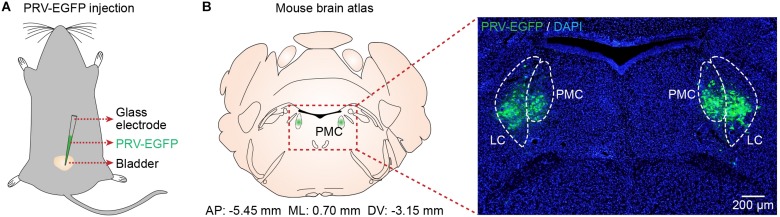
Verification of PMC location. **(A)** Schematic of PRV-EGFP injection into the bladder walls of mice. **(B)** Left, the location of the PMC according to the mouse brain atlas. Right, confocal image showing PRV-infected neurons (green) in the PMC with DAPI (blue) staining

To identify the location of the PMC, we used PRV-based retrograde trans-synaptic tracing (Smith et al., 2000; Stanley et al., 2010). Immunohistochemical analyses of brain slices 4.5 to 5 days after PRV-EGFP injection into the bladder walls of mice (*n* = 5) (Figure 1A) revealed PRV-infected neurons in the PMC and LC according to the mouse brain atlas (Figure 1B), as reported recently (Yao et al., 2018). Therefore, we used this identified location of the PMC for the following recordings.

### Population Ca^2+^ Transients of PMC Neurons Highly Correlate With Urination Events in Freely Moving Mice

**FIGURE 2:**
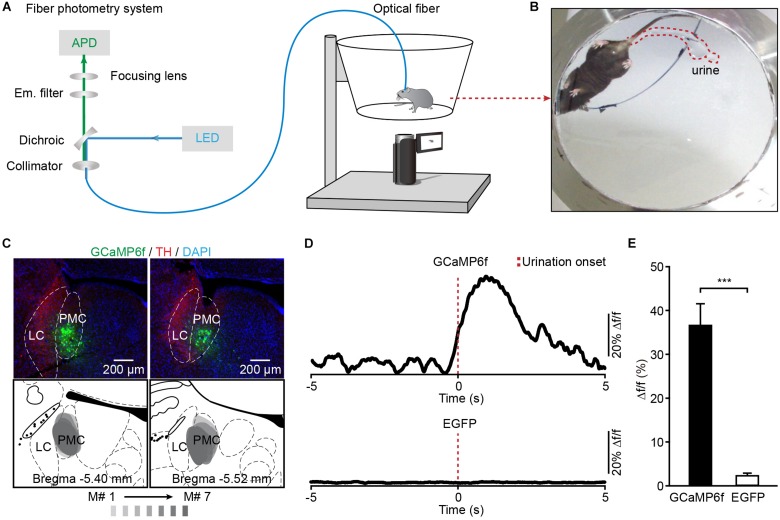
Population Ca^2+^ transients of PMC neurons in freely moving mice during urination. **(A)** Schematic of the fiber photometry setup. **(B)** The example picture shows that the mouse behavior and urine deposition were monitored using a camera placed underneath the chamber. **(C)** The representative *post hoc* images showing AAV-hSyn-GCaMP6f-labeled neurons (green) in the PMC. LC neurons are labeled with TH (red). Serial brain sections showing overlay of viral expression areas (in gray) from 7 GCaMP6f-injected mice. **(D)** Examples of Ca^2+^ transients of PMC neurons during urination events in the GCaMP6f-injected group (upper) and the EGFP-injected group (down). The red dashed line indicates urination onset. **(E)** Quantification of amplitudes of urination-related Ca^2+^ transients in the GCaMP6f-injected group (*n* = 7 mice) and the EGFP-injected group (*n* = 7 mice). Wilcoxon rank-sum test, ^∗∗∗^*P* < 0.001.

To investigate whether the activation of PMC neurons coincided with urination in freely moving mice, we monitored the Ca^2+^ activities of PMC neurons and urination events simultaneously. A genetically encoded Ca^2+^ indicator GCaMP6f was locally expressed in the PMC using a viral injection (AAV-hSyn-GCaMP6f). Fiber photometry was used to record population Ca^2+^ activity in the PMC. This optical fiber-based Ca^2+^ recording system (Figure 2A) was described in our previous work (Zhang et al., 2017; Qin et al., 2018; Yao et al., 2018). Each mouse was put in the chamber, and its behavior was visualized using a camera placed under the chamber (Figures 2A,B). An optical fiber with a diameter of 200 μm was implanted above GCaMP6f-positive PMC neurons 1 month after the viral injection (Figure 2C). PMC neurons were near the LC, which was identified using the tyrosine hydroxylase (TH) expression (Figure 2C). Serial sectioning also confirmed that GCaMP6f-positive neurons were largely restricted to the PMC area in all the GCaMP6f-injected mice reported here (*n* = 7) (Figure 2C). To increase the number of urination events, each mouse was treated with an intraperitoneal (i.p.) injection of diuretic furosemide (40 mg/kg) or 2 mL saline prior to recording. A representative example revealed that Ca^2+^ transients of PMC neurons were observed in the GCaMP6f-injected group during urination (Figure 2D). These urination-related transients were not observed in the EGFP-injected control group (Figures 2D,E; GCaMP6f-injected group, max Δf/f = 36.73% ± 4.90%; EGFP-injected group, max Δf/f = 2.24% ± 0.66%; *P* < 0.001). These experiments indicated that the Ca^2+^ transients of the PMC in the GCaMP6f-injected group were not movement artifacts.

**FIGURE 3:**
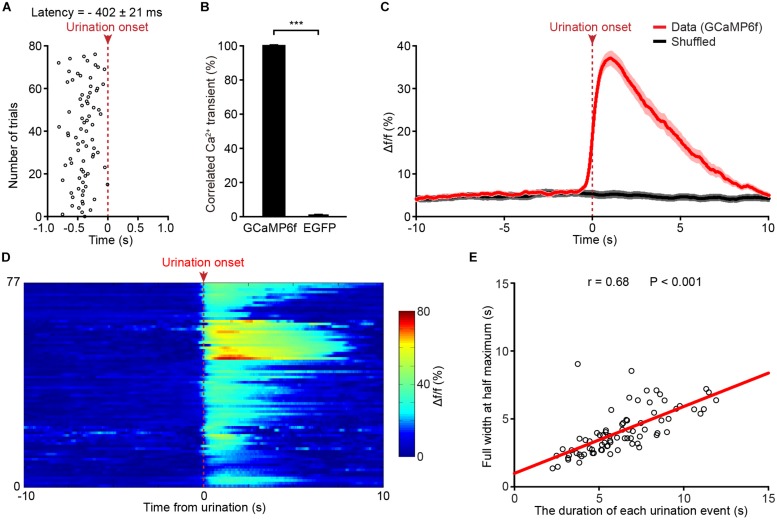
Population Ca^2+^ transients of PMC neurons highly correlate with urination events in freely moving mice. **(A)** Onsets of PMC Ca^2+^ transients in the GCaMP6f-injected group plotted relative to the onset of urination (red dashed line). *n* = 77 urination events from 7 mice. **(B)** Quantification of the percentage of Ca^2+^ events that correlated with urination in the GCaMP6f-injected group and the EGFP-injected group. Wilcoxon rank-sum test, ^∗∗∗^*P* < 0.001. **(C)** Averaged Ca^2+^ transients of the PMC neurons in the GCaMP6f-injected group (red, *n* = 77 urination events from 7 mice) aligned to the urination onset or shuffled urination onset (red dashed line). The dashed bar indicates urination onset. Shaded areas represent s.e.m. **(D)** Heatmap showing Ca^2+^ transients of the PMC neurons aligned to urination onsets in freely moving mice. *n* = 77 urination events from 7 mice. Warmer color indicates higher amplitude of urination-related Ca^2+^ transients. **(E)** Quantification of the full width at the half maximum of Ca^2+^ transients in D in relation to the duration of urination events. The duration of each urination event was measured from the urination onset (the first drop of urine appearing at the urethral orifice) to the urination offset (no drop of urine appearing at the urethral orifice).

A closer analysis showed that Ca^2+^ transients of PMC neurons in the GCaMP6f-injected group started 402 ± 21 ms prior to the onset of urination events (Figure 3A). We found that each urination event was 100% associated with Ca^2+^ transients in the GCaMP6f-injected group throughout all the recordings (Figure 3B; *P* < 0.001). Ca^2+^ transients of PMC GCaMP6f-positive neurons started before urination onset on average and in single urination events (heat map), and this response was absent in shuffled data (Figures 3C,D). Further analyses indicated that the duration of urination events linearly correlated to the full width at the half maximum of Ca^2+^ transients (Figure 3E). Taken together, neuronal activities in the PMC highly correlated with urination.

### Simultaneous Recording of Neuronal Activity in the PMC and Cystometry in Freely Moving Mice

**FIGURE 4:**
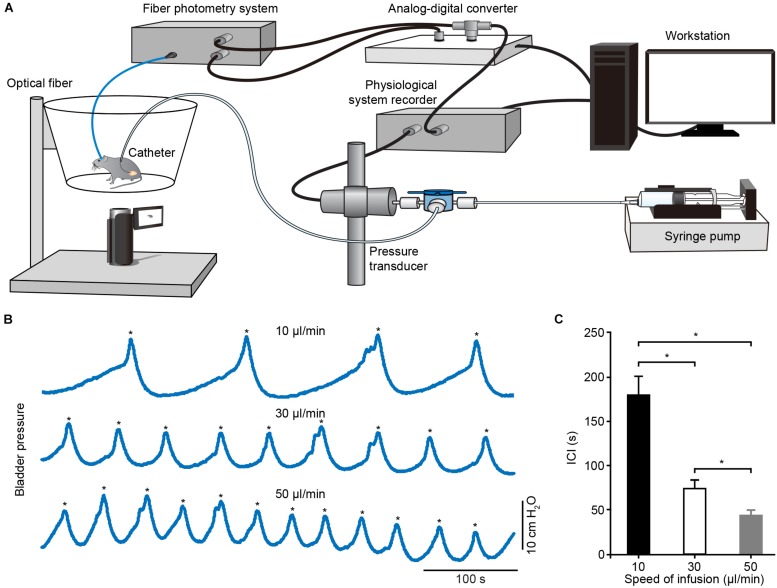
Schematic of experimental recording setup and cystometrogram. **(A)** Schematic of the urodynamic setup incorporating the fiber photometry system in freely moving mice. **(B)** Examples showing the bladder pressures in an anesthetized mouse at different infusion speeds. The voiding contractions were pointed out with the asterisks. **(C)** Quantitative analysis of the ICI at different infusion speeds. *n* = 6 mice. Wilcoxon signed-rank test, ^*^P < 0.05.

To determine whether Ca^2+^ transients of PMC neurons correlated to bladder detrusor contraction, we simultaneously monitored population Ca^2+^ transients of PMC GCaMP6f-positive neurons and urinary bladder pressure in freely moving mice. The experimental setup included a custom-built fiber setup and cystometry system (Figure 4A). Bladder pressure was measured using cystometry via a catheter inserted into the mouse bladder. Representative traces of intravesical pressures were recorded in an urethane-anesthetized mouse at different bladder filling speeds (Figure 4B). Each voiding contraction was represented as a spike in the intravesical pressure followed by a drop to the lowest pressure and associated with urination (indicated with asterisks in Figure 4B), reflecting urination and empty bladder. The intercontractile interval (ICI) was the duration between two adjacent voiding contractions. Quantitative analyses demonstrated that the ICI was tightly dependent on the level of infusion speed (*n* = 6 mice) (Figure 4C; 10 μl/min, 179.20 s ± 21.25 s; 30 μl/min, 74.18 s ± 10.76 s; 50 μl/min, 43.38 s ± 5.94 s; *P* < 0.05). As previously reported (Hou et al., 2016), we infused saline into the bladder slowly at a constant speed, which steadily triggered spike-like bladder voiding contractions.

**FIGURE 5:**
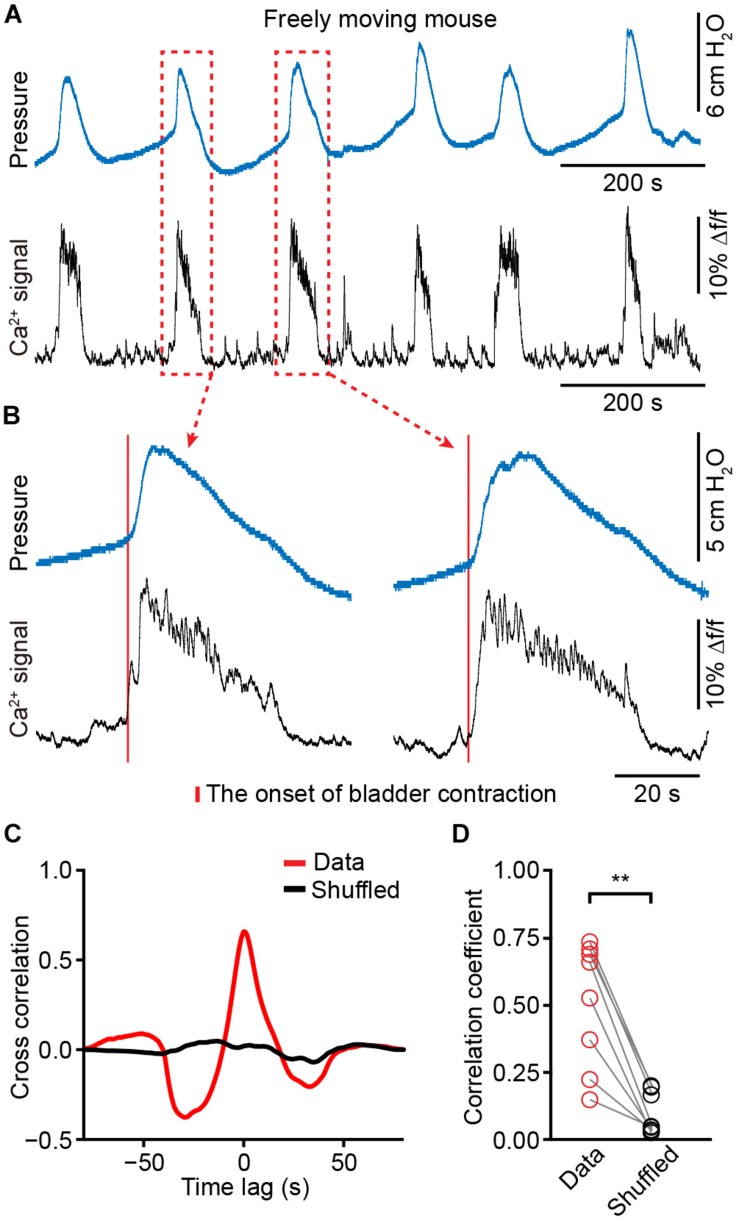
The activities of PMC neurons highly correlate with increased bladder pressure in freely moving mice. **(A)** Example bladder pressure trace (blue) and time-locked Ca^2+^ transients of PMC neurons (black) in a freely moving mouse. **(B)** The enlarged view of the dashed line boxes from A. The red bar indicates the onset of bladder contraction. **(C)** Cross-correlation between Ca^2+^ transients of PMC neurons and bladder pressure from the awake mouse as shown in A compared to the shuffled data. **(D)** Summary of cross-correlation coefficients. *n* = 8 mice. Wilcoxon signed-rank test, ^∗∗^*P* < 0.01.

We placed a catheter into the bladder of each animal in which fiber photometry was successfully recorded (*n* = 8 mice). Mice recovered for at least 5 days, and simultaneous measurement of neuronal activity and cystometry showed that spike-like increases in bladder pressure (bladder contraction) correlated with Ca^2+^ transients of PMC neurons in freely moving mice (Figure 5A). The expanded graphs in Figure 5B of two urination cycles indicate that the Ca^2+^ transients preceded the onset of bladder contraction. Quantitative analyses showed that the onset of Ca^2+^ transients in the PMC was ∼125 ± 66 ms earlier than bladder contraction (*n* = 8 mice). We also found that each spike-like increase in bladder pressure was also 100% associated with Ca^2+^ transients of PMC GCaMP6f-positive neurons. Cross-correlation of Ca^2+^ transients recorded in the PMC and bladder pressure showed that the correlation coefficient of the mean peak value was higher than the shuffled data (*n* = 8 mice) (Figures 5C,D; data, 0.51 ± 0.08; shuffled, 0.09 ± 0.03; *P* < 0.01). These results demonstrate that population Ca^2+^ transients of PMC neurons correlate with bladder contraction in freely moving mice.

### Anesthesia Evokes Prominent Changes in the Dynamics of Ca^2+^ Signals in the PMC During the Bladder Contraction

**FIGURE 6:**
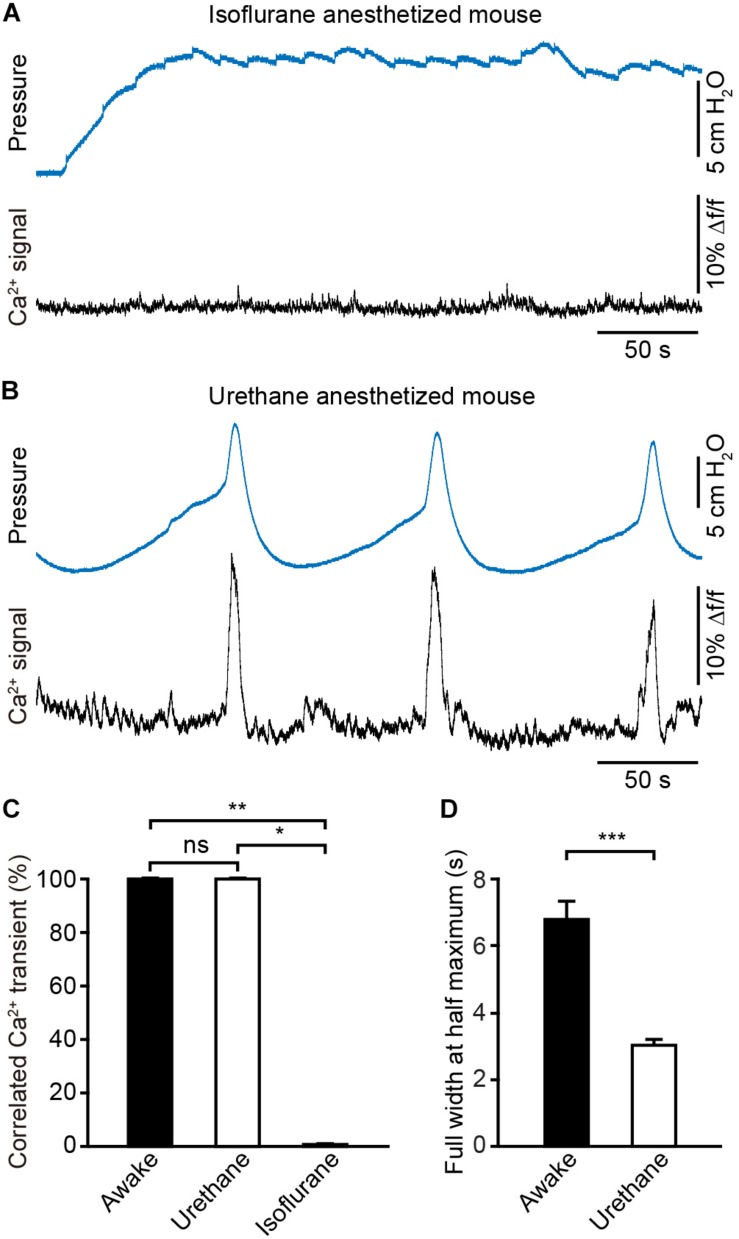
Anesthesia evokes prominent changes in the dynamics of Ca^2+^ signals in the PMC during the bladder contraction. **(A)** Example bladder pressure trace (blue) and time-locked Ca^2+^ transients of PMC neurons (black) in an isoflurane-anesthetized mouse. **(B)** Example bladder pressure trace (blue) and time-locked Ca^2+^ transients of PMC neurons (black) in a urethane-anesthetized mouse. The mouse as shown in A and B was the same one. **(C)** Quantification of the percentage of Ca^2+^ events that correlated with the bladder contractions. *n* = 4 mice for anesthesia and 8 mice for wakefulness. Wilcoxon rank-sum test, ^∗∗^*P* < 0.01 or Wilcoxon signed-rank test, ^*^P < 0.05. **(D)** Quantitative analysis of the full width at the half maximum of Ca^2+^ transients in each group. *n* = 40 trials from 4 awake mice; *n* = 44 trials from 4 urethane-anesthetized mice. Wilcoxon rank-sum test, ^∗∗∗^*P* < 0.001.

To assess the effect of anesthesia on the neuronal activity in the PMC, we simultaneously monitored population Ca^2+^ transients of PMC GCaMP6f-positive neurons and urinary bladder pressure in anesthetized mice (*n* = 4). Firstly, these catheter-implanted mice that were successfully recorded with fiber photometry were anesthetized with 2.5% isoflurane. We found that bladder contraction-related population Ca^2+^ transients of PMC neurons were completely absent under the isoflurane-anesthetized condition (Figure 6A). Cystometric recordings revealed that the regular urination cycles were not detected due to dripping overflow incontinence in this condition (Figure 6A).

Then isoflurane was withdrawn and anesthesia was maintained with urethane. Under this condition mice urinated periodically and simultaneous measurement of neuronal activity and cystometry showed that bladder contraction correlated with Ca^2+^ transients of PMC neurons (Figure 6B). Each spike-like increase in bladder pressure was also 100% associated with Ca^2+^ transients of PMC neurons in the urethane-anesthetized group (*n* = 4 mice), the same as what in the awake group (*n* = 8 mice) (Figure 6C). The dynamics of Ca^2+^ transients of PMC neurons were significantly different between awake and urethane-anesthetized mice (Figures 5A, 6B). Quantitative analyses demonstrated that the full width at the half maximum of Ca^2+^ transients in the urethane-anesthetized group was smaller than that in the awake group (Figure 6D; the urethane-anesthetized group, 3.02 ± 0.19 s; the awake group, 6.77 ± 0.57 s; *P* < 0.001). The normal saline was infused into the mouse bladders via the catheter at 30 μl/min in each trial of the two groups (*n* = 40 trials from 4 awake mice; *n* = 44 trials from 4 urethane-anesthetized mice). Overall, these results indicate that urethane anesthesia evokes prominent changes in the dynamics of the Ca^2+^ signals in the PMC, and deep isoflurane anesthesia induces the dripping overflow incontinence which may be due, at least in part, due to suppression of the neural activity in the PMC.

## Discussion

The present study combined optical fiber-based Ca^2+^ recording with cystometry to examine how neural activities of PMC neurons were related to urination or bladder pressure in freely moving mice. We found that population Ca^2+^ transients of PMC neurons appeared during each bladder contraction by cystometry. We observed that the onset of Ca^2+^ transients in the PMC was ∼400 ms earlier than the onset of urination events or ∼125 ms earlier than bladder contraction. In addition, a persistent class of Ca^2+^ transients of PMC neurons was seen throughout the entire process of urination. Finally, we showed that urethane anesthesia evoked prominent changes in the dynamics of Ca^2+^ signals in the PMC and deep isoflurane anesthesia induced the dripping overflow incontinence due to suppression of the neural activity in the PMC.

Urodynamic measurements in humans are mostly collected under a fully awake state (Gammie et al., 2014). Previous studies have reported that anesthesia modifies bladder function (Matsuura and Downie, 2000; Chang and Havton, 2008; Wuethrich et al., 2013; Aizawa et al., 2015; Schneider et al., 2015). Our results also showed that anesthesia evoked prominent changes in the dynamics of the Ca^2+^ signals in the PMC during the bladder contraction and even induced the dripping overflow incontinence. Therefore, the performance of cystometry in fully awake animals would be a promising translational measurement to evaluate bladder function. However, most previous reports that recorded the neuronal activity of PMC neurons and bladder pressure simultaneously were performed in anesthetized animals (Tanaka et al., 2003; de Groat and Wickens, 2013) and no data are available for simultaneous recordings in freely moving animals. Therefore, our measurements in freely moving mice are a new and efficient approach to investigate brain functions during urination.

Cystometry is widely used to monitor the bladder pressure and micturition cycles (Griffiths, 1977; Fry et al., 2010). However, it requires chronic catheter implantation into the bladder, which causes a local bladder tissue reaction, bladder wall swelling and post-operative stress (Yaksh et al., 1986; Mann-Gow et al., 2017). Recent work compared the bladder function in the same animals before and after catheter implantation surgery and showed that the catheter implantation operation did not affect bladder function (Schneider et al., 2018). The skills of the experimenter and the amount of recovery time after catheter implantation surgery were critical for the quality and reproducibility of results. Bladder wall swelling and voiding frequency were normal at least 5 days after surgery (Mann-Gow et al., 2017). Therefore, we used a microsurgical technique that minimized bladder damage during catheter implantation surgery (Mann-Gow et al., 2017) and offered the mice at least 5 days for recovery. In addition, this measurement could not completely mimic physiological micturition cycles, which are less frequent when saline is not infused into the bladder (Manohar et al., 2017). Notably, our present findings showed that the pattern of population neuronal activities in the PMC during natural micturition cycles was similar to the micturition cycles using cystometry.

Three types of activity patterns of PMC neurons were distinguished in several reports (Tanaka et al., 2003; de Groat and Wickens, 2013; de Groat et al., 2015): (1) Some neurons fired before and during bladder contractions; (2) some neurons were suppressed during bladder contractions and activated during the storage period; and (3) other neurons only discharged at the beginning of the detrusor contractions. However, our present findings only showed that the neuronal activity of PMC neurons started prior to bladder contractions and persisted throughout the voiding in freely moving mice. This difference may be because the fiber photometry-detected Ca^2+^ transients reflected the average spiking activity of all types of PMC neurons. The rapid development of Ca^2+^ imaging techniques suggests that a micro-endoscopic approach would record population Ca^2+^ transients in any brain region of freely moving animals at single-cell resolution (Jennings et al., 2015; Resendez et al., 2016). The application of this method would further our understanding of the properties of urination-related signals of PMC neurons.

Fiber photometry is an increasingly used population method for the monitoring of population activities of specific neurons in freely moving animals, including mice and monkeys (Adelsberger et al., 2014; Gunaydin et al., 2014). Several recent studies used this method to investigate urination-related neuronal population activities in freely moving mice. Hou et al. (2016) reported urination-related activities from a cluster of neurons expressing corticotropin-releasing hormone (CRH) that modulated the bladder contraction, and Keller et al. (2018) found similar activities in a group of neurons expressing estrogen receptor 1 (ESR1), which controlled sphincter relaxation. More recently, we used fiber photometry and found voluntary urination-related activities in mouse primary motor cortex (Yao et al., 2018). These activities are required for the driving of urination through the projections to the PMC. Therefore, urodynamic measurement that incorporate the optical fiber-based Ca^2+^ recording in freely moving mice will be an efficient approach for investigating the brain circuitry that controls urination.

In summary, our results revealed that a urodynamic measurement incorporating the optical fiber-based Ca^2+^ recording was an efficient approach for the detection of population neuronal activities of any brain region during urination in freely moving mice. We provide a better insight into the neural mechanisms that control the bladder and open up a possible avenue to elucidate NGB using a translational method.

## Ethics Statement

This study was approved by the Institutional Animal Care and Use Committee of Third Military Medical University. All experimental procedures were conducted in accordance with animal ethical guidelines of the Third Military Medical University Animal Care and Use Committee.

## Author Contributions

JiY, XC, WL, and JuY contributed to the design of the study and interpretation of the data. JiY, QL, XpL, and HQ carried out the experiments and acquired the data. JiY, XL, SL, and HQ processed and analyzed the data. JiY, XC, WL, and JuY wrote the manuscript with help from all other authors.

## Conflict of Interest Statement

The authors declare that the research was conducted in the absence of any commercial or financial relationships that could be construed as a potential conflict of interest.

## Footnotes

1 https://github.com/xieyangshuying/FRC
